# Age-related differences in the survival benefit of the administration of antithrombin, recombinant human thrombomodulin, or their combination in sepsis

**DOI:** 10.1038/s41598-022-13346-3

**Published:** 2022-06-03

**Authors:** Takeshi Wada, Kazuma Yamakawa, Daijiro Kabata, Toshikazu Abe, Hiroshi Ogura, Atsushi Shiraishi, Daizoh Saitoh, Shigeki Kushimoto, Seitaro Fujishima, Toshihiko Mayumi, Toru Hifumi, Yasukazu Shiino, Taka-aki Nakada, Takehiko Tarui, Yasuhiro Otomo, Kohji Okamoto, Yutaka Umemura, Joji Kotani, Yuichiro Sakamoto, Junichi Sasaki, Shin-ichiro Shiraishi, Kiyotsugu Takuma, Ryosuke Tsuruta, Akiyoshi Hagiwara, Tomohiko Masuno, Naoshi Takeyama, Norio Yamashita, Hiroto Ikeda, Masashi Ueyama, Satoshi Fujimi, Satoshi Gando

**Affiliations:** 1 Division of Acute and Critical Care Medicine, Department of Anesthesiology and Critical Care Medicine, Hokkaido University Faculty of Medicine, Kita-ku, Sapporo, N15, W7 Japan; 2 Department of Emergency Medicine, Osaka Medical and Pharmaceutical University, Takatsuki, Japan; 3 Department of Medical Statistics, Osaka City University Graduate School of Medicine, Osaka, Japan; 4 Department of Emergency and Critical Care Medicine, Tsukuba Memorial Hospital, Tsukuba, Japan; 5 Health Services Research and Development Center, University of Tsukuba, Tsukuba, Japan; 6 Department of Traumatology and Acute Critical Medicine, Osaka University Graduate School of Medicine, Suita, Japan; 7 Emergency and Trauma Center, Kameda Medical Center, Kamogawa, Japan; 8 Division of Traumatology, Research Institute, National Defense Medical College, Tokorozawa, Japan; 9 Division of Emergency and Critical Care Medicine, Tohoku University Graduate School of Medicine, Sendai, Japan; 10 Center for General Medicine Education, Keio University School of Medicine, Tokyo, Japan; 11 Department of Emergency Medicine, School of Medicine, University of Occupational and Environmental Health, Kitakyushu, Japan; 12 Department of Emergency and Critical Care Medicine, St. Luke’s International Hospital, Tokyo, Japan; 13 Department of Acute Medicine, Kawasaki Medical School, Kurashiki, Japan; 14 Department of Emergency and Critical Care Medicine, Chiba University Graduate School of Medicine, Chiba, Japan; 15 Department of Emergency Medical Care, Kyorin University Faculty of Health Sciences, Mitaka, Japan; 16 Trauma and Acute Critical Care Center, Medical Hospital, Tokyo Medical and Dental University, Tokyo, Japan; 17 Department of Surgery, Center for Gastroenterology and Liver Disease, Kitakyushu City Yahata Hospital, Kitakyushu, Japan; 18 Division of Trauma and Surgical Critical Care, Osaka General Medical Center, Osaka, Japan; 19 Division of Disaster and Emergency Medicine, Department of Surgery Related, Kobe University Graduate School of Medicine, Kobe, Japan; 20 Emergency and Critical Care Medicine, Saga University Hospital, Saga, Japan; 21 Department of Emergency and Critical Care Medicine, Keio University School of Medicine, Tokyo, Japan; 22 Department of Emergency and Critical Care Medicine, Aizu Chuo Hospital, Aizu, Japan; 23 Emergency & Critical Care Center, Kawasaki Municipal Hospital, Kawasaki, Japan; 24 Advanced Medical Emergency & Critical Care Center, Yamaguchi University Hospital, Ube, Japan; 25 Center Hospital of the National Center for Global Health and Medicine, Tokyo, Japan; 26 Department of Emergency and Critical Care Medicine, Nippon Medical School, Tokyo, Japan; 27 Advanced Critical Care Center, Aichi Medical University Hospital, Nagakute, Japan; 28 Department of Emergency and Critical Care Medicine, School of Medicine, Kurume University, Kurume, Japan; 29 Department of Emergency Medicine, Trauma and Resuscitation Center, Teikyo University School of Medicine, Tokyo, Japan; 30 Department of Trauma, Critical Care Medicine, and Burn Center, Japan Community Healthcare Organization, Chukyo Hospital, Nagoya, Japan; 31 Department of Acute and Critical Care Medicine, Sapporo Higashi Tokushukai Hospital, Sapporo, Japan

**Keywords:** Coagulation system, Bacterial infection

## Abstract

Disseminated intravascular coagulation (DIC) is one of the major organ dysfunctions associated with sepsis. This retrospective secondary analysis comprised data from a prospective multicenter study to investigate the age-related differences in the survival benefit of anticoagulant therapy in sepsis according to the DIC diagnostic criteria. Adult patients with severe sepsis based on the Sepsis-2 criteria were enrolled and divided into the following groups: (1) anticoagulant group (patients who received anticoagulant therapy) and (2) non-anticoagulant group (patients who did not receive anticoagulant therapy). Patients in the former group were administered antithrombin, recombinant human thrombomodulin, or their combination. The increases in the risk of hospital mortality were suppressed in the high-DIC-score patients aged 60–70 years receiving anticoagulant therapy. No favorable association of anti-coagulant therapy with hospital mortality was observed in patients aged 50 years and 80 years. Furthermore, anticoagulant therapy in the lower-DIC-score range increased the risk of hospital mortality in patients aged 50–60 years. In conclusion, anticoagulant therapy was associated with decreased hospital mortality according to a higher DIC score in septic patients aged 60–70 years. Anticoagulant therapy, however, was not associated with a better outcome in relatively younger and older patients with sepsis.

## Introduction

Disseminated intravascular coagulation (DIC) is characterized by systemic thrombin generation, not restricted to the site of insult, and is followed by microvascular fibrin thrombosis. Since the 1990s, DIC has been known as one of the major organ failures associated with sepsis^1,2^. Moreover, DIC gives rise to multiple organ dysfunction and affects patient outcomes^3^. Large-scale randomized clinical trials (RCTs) have been conducted to verify the effects of anticoagulant agents, including the HETRASE (Unfractioned Heparin for Treatment of Sepsis) study for unfractionated heparin^4^; the KyberSept trial for antithrombin^5^; the PROWESS (Recombinant Human Activated Protein C Worldwide Evaluation in Severe Sepsis), ADDRESS (Administration of Drotrecogin Alfa in Early Stage Severe Sepsis), and PROWESS-SHOCK trials for activated protein C^6–8^; the OPTIMIST (The Optimized Phase 3 Tifacogin in Multicenter International Sepsis Trial) and CAPTIVATE (Community-Acquired Pneumonia Tifacogin Intra-Venous Administration Trial for Efficacy) for tissue factor pathway inhibitor^9,10^; and the SCARLET (Sepsis Coagulopathy Asahi Recombinant LE Thrombomodulin) trial for recombinant human thrombomodulin^11^. However, these aforementioned studies failed to determine the efficacy of anticoagulant therapy against sepsis, which may be attributed to the fact that most of these RCTs targeted heterogeneous patients, such as those with “sepsis” or “severe sepsis”^12^. Recently, it has become widely apparent that patients with three factors, namely, “sepsis,” “DIC,” and “high disease severity,” may constitute an optimal target for anticoagulant therapies^13^. We previously demonstrated that anticoagulant therapy is associated with better outcomes according to the deterioration of both DIC and disease severity^14^, which were evaluated by the International Society on Thrombosis and Haemostasis (ISTH) overt DIC scoring system^1^ and the Acute Physiology and Chronic Health Evaluation (APACHE) II score, respectively^15^.

The ISTH overt DIC diagnostic criteria have sufficient accuracy for diagnosing DIC. However, it has been pointed out that by the time overt DIC can be identified by the ISTH criteria, the patient may already be in an irreversible and decompensated stage, which may be too late, from a therapeutic perspective, for initiating effective interventions^3^. The Japanese Association for Acute Medicine (JAAM) DIC diagnostic criteria, which were established to overcome the limitations of the ISTH overt DIC criteria, have good diagnostic properties and predictive accuracy for 28-day and hospital mortality in patients with severe sepsis^16^. In addition, the JAAM DIC diagnostic criteria can detect twice as many cases in less time than that needed by the ISTH overt DIC criteria^17^. The APACHE II scoring system, which is a classification system for measuring disease severity, comprises three components: age, chronic disease score, and the Acute Physiology Score (APS). APACHE II score has been widely used to predict outcomes, including sepsis, for critically ill patients^18^. However, the APS is defined as a sum of the worst values of 12 physiological indicators within 24 h after intensive care unit (ICU) admission; thus, considering its simplicity and promptness, the APACHE II score may not be useful for decision-making at the time of treatment initiation. Thus, based on previous evidence that DIC is simultaneously associated with increasing disease severity, we focused on age, which is one of the components of the APACHE II score. Aging is accompanied by an increase in the prothrombotic state^19^, suggesting that age may be a key factor in the pathophysiology of sepsis-associated DIC, affecting the efficacy of anticoagulant therapy against sepsis.

Therefore, this study was conducted, using the nationwide sepsis registry data set, with the aim of examining the age-related differences in the survival benefit conferred by anticoagulant therapy, defined as the administration of antithrombin, recombinant human thrombomodulin, or their combination in sepsis in accordance with the JAAM DIC diagnostic criteria.

## Results

### Study population

**Figure 1:**
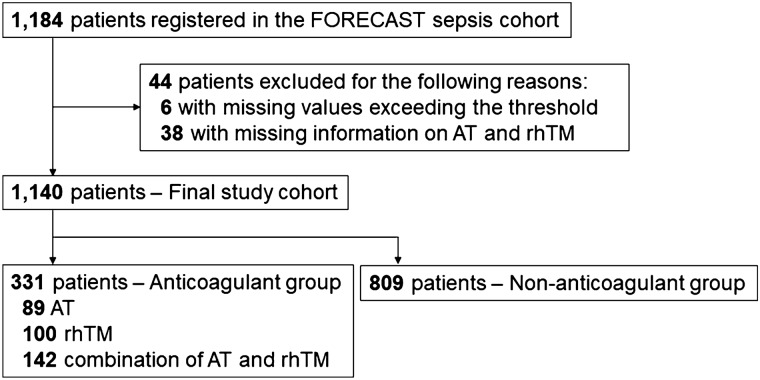
Flow chart of the study population. AT, antithrombin; FORECAST, Focused Outcomes Research in Emergency Care in Acute Respiratory Distress Syndrome, Sepsis and Trauma; rhTM, recombinant human thrombomodulin.

In total, 1184 consecutive patients fulfilling the inclusion criteria were registered during the study period in the JAAM Focused Outcomes Research in Emergency Care in Acute Respiratory Distress Syndrome, Sepsis and Trauma (FORECAST) Sepsis cohort. Six registered patients who had missing values exceeding the threshold (> 170) were detected by a one-sample robust regression with an M estimator. Moreover, 38 patients were excluded due to missing information on the administration of antithrombin and recombinant human thrombomodulin. Subsequently, the data from a final cohort of 1140 patients were analyzed in the present study. The anticoagulant group comprised 331 patients (antithrombin, 89 patients; recombinant human thrombomodulin, 100; and their combination, 142), and the non-anticoagulant group comprised 809 patients (Fig. 1).

Table 1 shows the baseline clinical characteristics and therapeutic interventions administered to the patients with or without anticoagulant treatments. Patient characteristics, such as age and sex, were similar in the two groups. The Charlson Comorbidity Index (CCI), APACHE II, Sequential Organ Failure Assessment (SOFA), and DIC scores were significantly higher in the anticoagulant group than in the non-anticoagulant group. In addition, the anticoagulant group showed higher rates of septic shock and positive blood culture than the non-anticoagulant group. The commonest sites of infection were the lung in the non-anticoagulant group and abdomen in the anticoagulant group, and these rates differed between the two groups. The rates of concomitant therapeutic interventions were also different between the two groups.

**Table 1:** Baseline clinical characteristics of the patients who did or did not receive anticoagulant therapy.

	Non-anticoagulant group N = 809	Anticoagulant group N = 331	*P*
**Patient characteristics**			
Age, years	73 (64–82)	72 (64–81)	0.609
Sex (female/male)	38.7/61.3 (313/496)	41.7/58.3 (138/193)	0.347
**Preexisting conditions**			
Charlson comorbidity index	1 (0–2)	1 (0–2)	0.017
ADL dependent/independent	25.1/74.9 (203/606)	21.2/78.8 (70/260)	0.164
Malignant disease, no/yes	87.4/12.6 (707/102)	83.4/16.6 (276/55)	0.075
Severe liver disease, no/yes	98.1/1.9 (794/15)	97.0/3.0 (321/10)	0.222
Prescribed anticoagulants, no/yes	90.6/9.4 (733/76)	91.2/8.8 (302/29)	0.737
**Illness severity**			
APACHE II score	21 (16–28)	27 (20–33)	< 0.001
SOFA score	8 (5–11)	10 (7–13)	< 0.001
SIRS score	3 (2–4)	3 (2–4)	0.176
ISTH DIC score	2 (1–4)	4 (3–5)	< 0.001
JAAM DIC score	3 (2–5)	5 (4–6)	< 0.001
Septic shock, no/yes	44.5/55.5 (360/449)	20.5/79.5 (68/263)	< 0.001
Blood culture, negative/positive	43.4/56.6 (349/455)	35.3/64.7 (117/214)	0.012
**Primary site of infection**			< 0.001
Abdomen	21.6 (175)	35.6 (118)	
Lung	35.6 (288)	20.5 (68)	
Urinary tract	18.8 (152)	19.6 (65)	
Skin/soft tissue	9.6 (78)	10.3 (34)	
Blood stream	2.1 (17)	1.2 (4)	
Bone/joint	2.0 (16)	1.2 (4)	
CNS	1.7 (14)	2.1 (7)	
Endocardium	1.4 (11)	1.5 (5)	
Implant device	0.9 (7)	0.3 (1)	
Wound	1.0 (8)	0.6 (2)	
Others	5.3 (43)	6.9 (23)	
**Therapeutic interventions**			
Mechanical ventilation, no/yes	52.2/47.8 (420/384)	42.4/57.6 (140/190)	0.003
PMX-DHP, no/yes	96.3/3.7 (779/30)	79.5/20.5 (263/68)	< 0.001
IVIg, no/yes	91.1/8.9 (733/72)	54.3/45.7 (178/150)	< 0.001
Protease inhibitor, no/yes	95.0/5.0 (768/40)	86.3/13.7 (283/45)	< 0.001
CRRT, no/yes	82.5/17.5 (664/141)	51.7/48.3 (171/160)	< 0.001
Corticosteroids, no/yes	77.4/22.6 (625/182)	51.2/48.8 (169/161)	< 0.001
Noradrenaline, no/yes	41.1/58.9 (332/475)	19.0/81.0 (63/268)	< 0.001
Enteral nutrition, no/yes	55.8/44.2 (450/357)	49.8/50.2 (164/165)	0.070

Data are presented as proportions (counts) for categorical variables and medians (interquartile ranges) for continuous variables. Anticoagulant therapy was defined as the administration of antithrombin, recombinant human thrombomodulin, or their combination in the present study.

ADL, activities of daily living; APACHE, Acute Physiology and Chronic Health Evaluation; CNS, central nervous system; CRRT, continuous renal replacement therapy; DIC, disseminated intravascular coagulation; IVIg, intravenous immunoglobulin; ISTH, International Society on Thrombosis and Haemostasis; JAAM, Japanese Association for Acute Medicine; PMX-DHP, polymyxin B direct hemoperfusion; SIRS, systemic inflammatory response syndrome; SOFA, Sequential Organ Failure Assessment.

### Effect of the administration of antithrombin, recombinant human thrombomodulin, or their combination on hospital mortality

**Figure 2:**
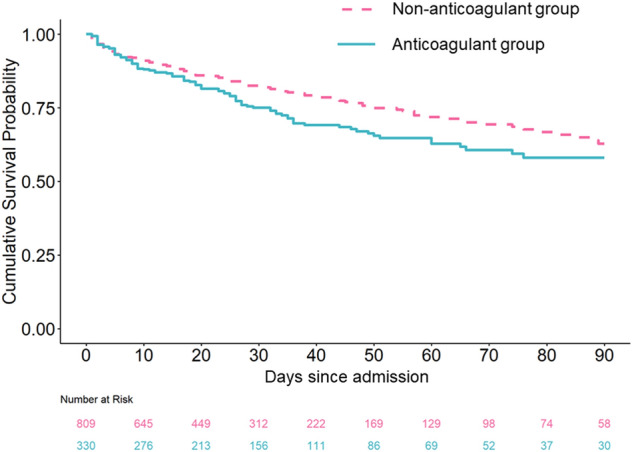
The Kaplan–Meier curves during the first 90 days for the cumulative survival of patients with and without anticoagulant therapy. The blue line represents the anticoagulant group and the dotted red line represents the non-anticoagulant group. Anticoagulant therapy was defined as the administration of antithrombin, recombinant human thrombomodulin, or their combination in the present study.

The overall cumulative survival probability that was obtained in all patients included in this study is shown in Fig. 2. The hospital mortality rate was 30.2% (100/331) in the anticoagulant group and 20.5% (166/809) in the non-anticoagulant group. The multivariable regression model showed that the hazard ratio (anticoagulant/non-anticoagulant) of hospital mortality was 0.910 (upper 0.665, lower 1.245, *P* = 0.554).

**Figure 3:**
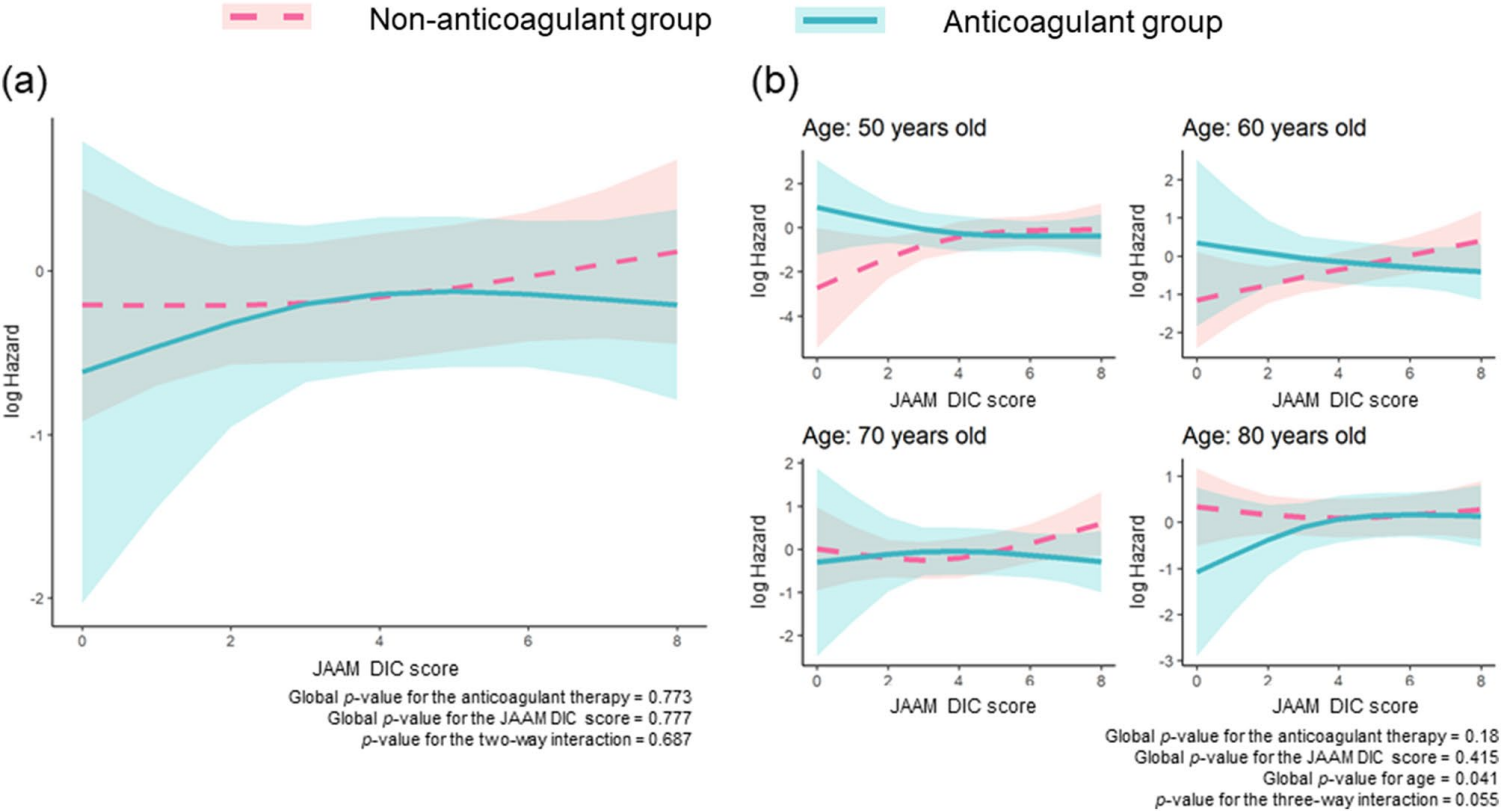
Regression line of hospital mortality of each treatment group estimated by the Cox proportional hazard regression model. (**a**) Two-way interaction term between the treatment and JAAM DIC score. (**b**) Three-way interaction term among anticoagulant therapy, the JAAM DIC score, and age. The lines indicate estimated log-transformed relative hazards, and the shaded areas represent 95% confidence intervals. The solid blue line represents patients in the anticoagulant group, and the dotted red line represents those in the non-anticoagulant group. JAAM, Japanese Association for Acute Medicine; DIC, disseminated intravascular coagulation. Anticoagulant therapy was defined as the administration of antithrombin, recombinant human thrombomodulin, or their combination in the present study.

We performed multivariate Cox proportional hazard regression model analysis, which included a two-way interaction, to identify the effect modification of anticoagulant therapy by the DIC score (Fig. 3a). Neither anticoagulant therapy nor the DIC score showed a significant interaction with hospital mortality (*P* = 0.773 and *P* = 0.777, respectively). The *P*-value for the two-way interaction was 0.687. The multivariate Cox proportional hazard regression model including the three-way interaction term between the previous two (anticoagulant therapy and DIC score) and age, which may influence the therapeutic effect, showed that the increases in the risk were suppressed in the population with higher DIC scores in patients aged 60 to 70 years in the anticoagulant group. In patients aged 50 years with sepsis, the risk in the non-anticoagulant group tended to increase concomitantly with increases in the DIC score in the low-score range (DIC score < 4), whereas no increase in the risk was observed in the high-score range (DIC score ≥ 4), and a favorable association of anticoagulant therapy with hospital mortality was not found. In patients aged 80 years, the non-anticoagulant group indicated a certain risk regardless of the DIC score, and the anticoagulant therapy showed no beneficial effect through a decreased risk of hospital mortality. Importantly, anticoagulant therapy in the lower DIC score range increased the risk hazard in patients aged 50 to 60 years. Global *P*-values for age and the three-way interaction were 0.041 and 0.055, respectively (Fig. 3b). The baseline clinical characteristics and therapeutic interventions in the four age groups (50 s, 60 s, 70 s, and 80 s) are shown in Supplementary Tables S1–S4.

### SOFA score 72 h after admission according to the DIC score

**Figure 4:**
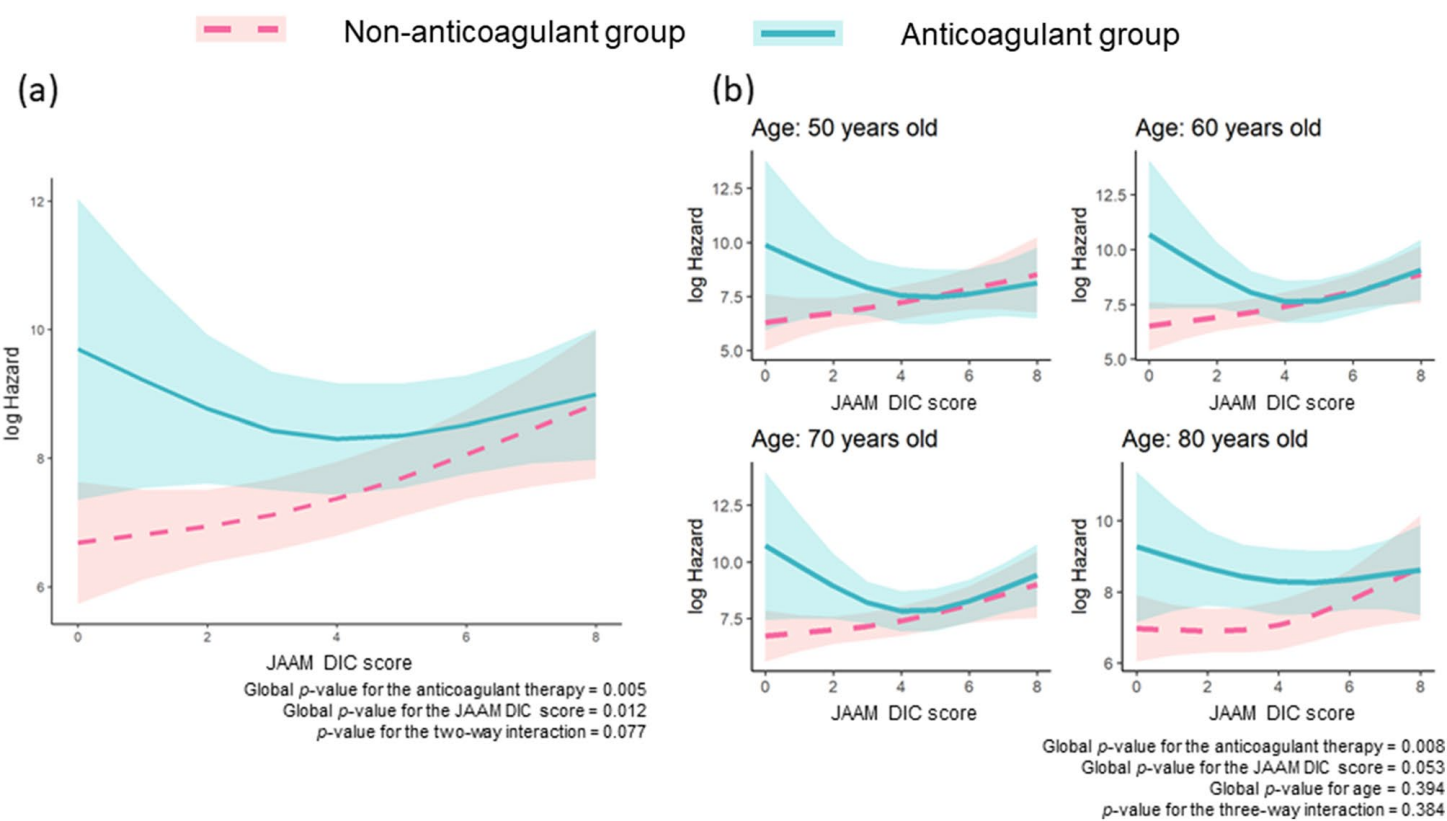
Regression lines of the SOFA score 72 h after admission in each treatment group. (**a**) Two-way interaction term between the treatment and JAAM DIC score. (**b**) Three-way interaction term among anticoagulant therapy, JAAM DIC score, and age. The lines indicate estimated log-transformed relative hazards, and the shaded areas represent 95% confidence intervals. The solid blue line represents patients in the anticoagulant group, and the dotted red line represents those in the non-anticoagulant group. Anticoagulant therapy was defined as the administration of antithrombin, recombinant human thrombomodulin, or their combination in the present study. JAAM, Japanese Association for Acute Medicine; DIC, disseminated intravascular coagulation; SOFA, Sequential Organ Failure Assessment.

The multivariate linear regression model was used to elucidate the effect modification of anticoagulant therapy on organ dysfunction by DIC score (Fig. 4). The risk hazard of the SOFA score 72 h after admission in the non-anticoagulant group increased concomitantly with an increase in DIC score. Anticoagulant therapy showed no favorable association with the SOFA score. In addition, when we considered the population with lower DIC scores, a higher risk of increase in the SOFA score was found in the anticoagulant group (Fig. 4a). We assessed the age-related effect of anticoagulant therapy on the SOFA score as well as on hospital mortality (Fig. 4b). Anticoagulant therapy did not improve the SOFA score in any age group, and it tended to induce a deterioration in SOFA scores in the low-DIC-score range.

## Discussion

We investigated the age-related differences in the survival benefit of anticoagulant therapy, defined as the administration of antithrombin, recombinant human thrombomodulin, and their combination in sepsis in accordance with the JAAM DIC diagnostic criteria. The results of this study indicate that anticoagulant therapy is associated with better outcomes consistent with a higher DIC score in patients aged 60 to 70 years. Moreover, anticoagulant therapy in patients with sepsis and low DIC scores may be associated with the deterioration of organ function and could lead to poor outcomes.

Previous studies have demonstrated that patients with sepsis and DIC exhibit an increased prevalence of multiple organ dysfunction and that the mortality rate of such patients is significantly higher than that of non-DIC patients^16,20^, suggesting that the development of DIC in sepsis is an indicator of poor prognosis. A recent nationwide multicenter retrospective cohort study suggested that screening for DIC was associated with a survival benefit in patients with sepsis^21^. These results imply that the evaluation and subsequent intervention for coagulofibrinolytic changes related to sepsis may contribute to improved outcomes in patients with sepsis. To date, however, there is no clear evidence that treating sepsis-induced coagulopathy leads to improved outcomes in patients with sepsis.

The important point is that most of the RCTs that evaluated the effect of anticoagulant therapy have targeted patients with “sepsis.” Although the KyberSept trial, a mega-RCT, demonstrated that antithrombin treatment did not affect 28-day mortality in adult patients with sepsis and septic shock^5^, its post hoc analysis suggested that antithrombin therapy was associated with a significant reduction in mortality only in patients with DIC but not in non-DIC patients^22^. These results indicate the importance of selecting the target population for anticoagulant therapy against sepsis. Studies showing that the optimal target for anticoagulant therapy is a patient population fulfilling three factors, namely, sepsis, DIC, and high disease severity, have recently been published^13,14,23–25^.

A recent global analysis demonstrated that 1 in 5 deaths around the world is caused by sepsis, leading to 11 million deaths annually worldwide, thus surpassing cancer mortality^26^. Older people constitute the major population of patients with sepsis, and mortality increases at more advanced ages^27,28^. Although it is well known that advancing age is associated with increased coagulation, this propensity for a deteriorating coagulation disorder during sepsis remains unknown^27^. Innate immune cells, such as neutrophils and macrophages, show age-related functional alteration, resulting in a diminished ability to rapidly respond to foreign pathogens^29^. In addition to the innate immune system, an attenuation of the adaptive immune functions in old age has been confirmed^30^. A prolonged inflammatory response in aged patients has been noted, as high levels of inflammatory cytokines have been found^31–34^. The interaction between the effect of anticoagulant therapy and the DIC score in patients aged 80 years that was found in our study may support these previous studies. In other words, the onset of sepsis itself may determine the outcome regardless of the severity of DIC in the elderly. In contrast, patients aged 50 years showed a different interaction between anticoagulant therapy and the DIC score. Relatively young patients with sepsis are expected to respond well to the treatment of underlying infection, followed by the modulation of dysregulated inflammatory and coagulofibrinolytic reactions. Therefore, it is presumed that the development of DIC in relatively young patients with sepsis may have minimal effect on the outcome, although the detailed underlying mechanisms remain unclear.

The present study failed to show a beneficial effect of the administration of antithrombin, recombinant human thrombomodulin, or their combination on the SOFA score of the population in any age group. However, this does not mean that organ dysfunction cannot be improved by anticoagulant therapy, because the SOFA scores were evaluated relatively early (72 h after admission). As this therapy improved hospital mortality in certain ages, it might have improved SOFA scores later than 72 h after admission. Our results should be considered when setting up the study designs of future RCTs to validate the effects of anticoagulant therapy against sepsis.

The main pathophysiology of DIC is uncontrolled thrombin generation, leading to ischemic organ dysfunction due to microvascular thrombosis, which is detrimental in the context of pathology. In contrast, the concept that microvascular thrombosis produced by innate immunity is a physiological process to maintain body homeostasis has come to be known as immunothrombosis^3,35^. From these perspectives, individuals who could be at an advantage with regard to immunothrombosis, namely, non-DIC patients, should not be treated with anticoagulant therapy. The harmful effects of anticoagulant therapy against non-DIC patients were confirmed in the present study (Figs. 3 and 4).

The current study has several limitations. First, although the present data set was prospectively collected, causal relationships could not be defined because of the study’s retrospective design. Second, this study did not assess the dosage and duration of anticoagulant agents. Additionally, this study was unable to evaluate the effects of individual anticoagulant agents or combination therapy and elucidate the potential effects of other anticoagulants, including heparin and serine protease inhibitors, because we defined anticoagulant therapy as the administration of antithrombin, recombinant human thrombomodulin, or their combination. Third, the efficacy of anticoagulant therapy might not have been evaluated correctly because we did not exclude patients with prescribed anticoagulants, similar to our previous study^14,36^. However, since the proportion of patients with prescribed anticoagulants was less than 10%, we believe that it might not have significantly affected the main results of this study. Fourth, the present data set did not include data on the administration of heparin, which is widely used as a prophylaxis for venous thromboembolism, and information on adverse effects, including serious bleeding complications associated with anticoagulant therapy, which can cause unfavorable outcomes. Fifth, data elements required to control potential confounders might have resulted in biased effect estimates. Finally, the study being conducted in a single country may limit the generalizability of the obtained results.

In conclusion, anticoagulant therapy, namely, the administration of antithrombin, recombinant human thrombomodulin, or their combination, showed a beneficial effect on hospital mortality according to a higher JAAM DIC score in patients aged 60 to 70 years with sepsis. Anticoagulant therapy, however, was not associated with a better outcome in relatively young (50 years) and older (80 years) patients. In addition, anticoagulant therapy in patients with low DIC scores was associated with the deterioration of organ function and poor outcomes, which may be caused by the destruction of the physiological hypercoagulative state and immunothrombosis. We suggest setting the inclusion criteria of future RCTs examining the effects of anticoagulant therapy against sepsis based on the results obtained from the present study.

## Methods

### Study design, setting, and ethical approval

This study was a retrospective secondary analysis of a sepsis cohort of the prospective, multicenter JAAM FORECAST study^36^. The JAAM FORECAST Sepsis study was conducted from January 2016 to March 2017 and used consecutive samples from 59 ICUs in Japan. The study was registered in the University Hospital Medical Information Network Clinical Trial Registry (UMIN-CTR ID: UMIN000019588). This study was approved by the JAAM and Ethics Committee of each hospital (JAAM, 2014-01; Hokkaido University Faculty of Medicine, head institute of the FORECAST group, 014-0307) after written informed consent was obtained from each patient or their next of kin and was performed in accordance with the tenets underlying the Declaration of Helsinki.

### Participants

The JAAM FORECAST Sepsis study enrolled adult patients (aged > 16 years) who had been admitted to the ICU with severe sepsis and septic shock according to the Sepsis-2 criteria published in 2003^37^, based on the following inclusion criteria: (i) patients suspected to have or who were diagnosed with new-onset infection based on the history of the present illness; (ii) patients who met ≥ 2 systemic inflammatory response syndrome (SIRS) criteria; and (iii) patients who had at least one organ dysfunction, defined by the following criteria: systolic blood pressure < 90 mmHg, mean arterial pressure < 65 mmHg, or decreased blood pressure > 40 mmHg from baseline; serum creatinine > 2.0 mg/dL or urine output < 0.5 mL/kg/h; total bilirubin > 2.0 mg/dL; platelet counts < 100 × 10^9^/L; lactate > 2 mmol/L; prothrombin time International Normalized Ratio (INR) > 1.5; and arterial hypoxemia (PaO_2_/FiO_2_) < 200 with pneumonia or PaO_2_/FiO_2_ < 250 without pneumonia. Patients on end-of-life care or those who were resuscitated following cardiac arrest at the time of the diagnosis of severe sepsis were excluded. Patients with substantial missing data were also excluded from the analysis. Patients with prescribed anticoagulants were not excluded, similar to our previous study, which used the same data set as the present study. The size of the study population was dependent on the study period. All patients were followed-up until discharge.

Participants were divided into two cohorts: the anticoagulant group and non-anticoagulant group. The anticoagulant group comprised patients who received anticoagulant therapy, defined as the administration of antithrombin, recombinant human thrombomodulin, or their combination, in accordance with the Japanese Clinical Practice Guidelines for Management of Sepsis and Septic Shock 2020^38^. The non-anticoagulant group comprised patients who received neither antithrombin nor recombinant human thrombomodulin. There was no pre-determined definitive protocol for the indication of anticoagulant therapy, which was initiated at the discretion of the participating physicians based on the institutional treatment policies at each hospital. The standard dosage of administration of antithrombin and recombinant human thrombomodulin for sepsis-induced DIC in Japan is 1,500 U/day or 30 U/kg/day, 3–5 days and 380 U/kg, 6 days, respectively.

### Definitions

We defined SIRS, sepsis, severe sepsis, and septic shock based on the American College of Chest Physicians/Society of Critical Care Medicine consensus conference (Sepsis-1) published in 1992^39^ and the revised version (Sepsis-2) published in 2003^37^. Disease severity was assessed according to the APACHE II score^15^. Organ dysfunction was evaluated according to the SOFA score^40^. The CCI was adopted for the assessment of baseline comorbidities^41^. DIC was diagnosed based on the JAAM DIC scoring system using prothrombin time INR as a substitute for the prothrombin time ratio (Supplementary Table S5)^42^.

### Data collection

An electronic data capture system was developed for use in this FORECAST study, and data compiled by the FORECAST investigators were obtained from the FORECAST database. Patient information included the baseline characteristics, various comorbidities, activities of daily living, suspected sites of infection, organ dysfunction, sepsis-related severity scores, and therapeutic interventions. Moreover, we obtained data on compliance with established sepsis care protocols, such as the measurement of serum lactate levels within 3 h of hospital arrival. The primary outcome was hospital all-cause mortality. The SOFA score 72 h after admission was recorded as the secondary outcome.

### Statistical analyses

All baseline clinical and demographical characteristics are expressed as medians and interquartile ranges for continuous variables, whereas numbers and percentages are used for categorical variables. For comparisons of these characteristics between the patients who were treated with and without anticoagulants, the Mann–Whitney *U* test and chi-square test were used for continuous and categorical variables, respectively.

To examine whether the effect of the anticoagulants on hospital mortality was modified by the DIC score, we used a multivariable Cox proportional hazard regression model that included a cross-product term between the anticoagulants’ variable and DIC score in addition to the main effect terms as the explanatory variables. The non-linear effect of the DIC score was assessed using a restricted-cubic-spline method with knot, k = 3. Furthermore, we assessed the patient’s age as the effect modifier using a similar regression model that included three- and two-way cross-product terms between the variables that indicated the effect of anticoagulants, including the DIC score and age. Moreover, similar analyses were performed for 72 h SOFA score as the explanatory variable using multivariable non-linear regression models, which were adjusted for all covariates that are described in Table 1. We confirmed that these regression models were not overfitted using bootstrap validation (index-corrected calibration slopes were approximately 0.8). The missing values were imputed using a multiple imputation method.

All tests were performed two-sided and significance level was set at 5%. Statistical analyses were performed using SPSS software version 26 (IBM Corp., Armonk, NY, USA) and R software version 4.1.1 (https://cran.r-project.org/).

## Supplementary Information

Supplementary Information.

## Data Availability

The data that support the findings of this study are available from the authors upon reasonable request.
