# Supplementary material for: Age-related differences in the survival benefit of the administration of antithrombin, recombinant human thrombomodulin, or their combination in sepsis
Source: Sci Rep. 2022 Jun 3;12:9304. doi: 10.1038/s41598-022-13346-3 (PMC9166729; doi:10.1038/s41598-022-13346-3)
Supplement: Supplementary file 1 — Supplementary Information. [file 41598_2022_13346_MOESM1_ESM.pdf]

Age-related differences in the survival benefit of the administration of antithrombin, recombinant human thrombomodulin, or their combination in sepsis

Takeshi Wada[1*], Kazuma Yamakawa[2], Daijiro Kabata[3], Toshikazu Abe[4,5], Hiroshi Ogura[6], Atsushi Shiraishi[7], Daizoh Saitoh[8], Shigeki Kushimoto[9], Seitaro Fujishima[10], Toshihiko Mayumi[11], Toru Hifumi[12], Yasukazu Shiino[13], Taka-aki Nakada[14], Takehiko Tarui[15], Yasuhiro Otomo[16], Kohji Okamoto[17], Yutaka Umemura[18], Joji Kotani[19], Yuichiro Sakamoto[20], Junichi Sasaki[21], Shin-ichiro Shiraishi[22], Kiyotsugu Takuma[23], Ryosuke Tsuruta[24], Akiyoshi Hagiwara[25], Tomohiko Masuno[26], Naoshi Takeyama[27], Norio Yamashita[28], Hiroto Ikeda[29], Masashi Ueyama[30], Satoshi Fujimi[18], Satoshi Gando[1,31]

[1]Division of Acute and Critical Care Medicine, Department of Anesthesiology and Critical Care Medicine, Hokkaido University Faculty of Medicine, Sapporo, Japan

[2]Department of Emergency Medicine, Osaka Medical and Pharmaceutical University, Takatsuki, Japan

[3]Department of Medical Statistics, Osaka City University Graduate School of Medicine, Osaka, Japan

[4]Department of Emergency and Critical Care Medicine, Tsukuba Memorial Hospital, Tsukuba, Japan

[5]Health Services Research and Development Center, University of Tsukuba, Tsukuba, Japan

[6]Department of Traumatology and Acute Critical Medicine, Osaka University Graduate School of Medicine, Suita, Japan

[7]Emergency and Trauma Center, Kameda Medical Center, Kamogawa, Japan

[8]Division of Traumatology, Research Institute, National Defense Medical College, Tokorozawa, Japan

[9]Division of Emergency and Critical Care Medicine, Tohoku University Graduate School of Medicine, Sendai, Japan

[10]Center for General Medicine Education, Keio University School of Medicine, Tokyo, Japan

[11]Department of Emergency Medicine, School of Medicine, University of Occupational and Environmental Health, Kitakyushu, Japan

[12]Department of Emergency and Critical Care Medicine, St. Luke's International Hospital, Tokyo, Japan

[13]Department of Acute Medicine, Kawasaki Medical School, Kurashiki, Japan

[14]Department of Emergency and Critical Care Medicine Chiba University Graduate School of Medicine, Chiba, Japan

[15]Department of Emergency Medical Care, Kyorin University Faculty of Health Sciences, Mitaka, Japan

[16]Trauma and Acute Critical Care Center, Medical Hospital, Tokyo Medical and Dental University, Tokyo, Japan

[17]Department of Surgery, Center for Gastroenterology and Liver Disease, Kitakyushu City Yahata Hospital, Kitakyushu, Japan

[18]Division of Trauma and Surgical Critical Care, Osaka General Medical Center, Osaka, Japan

[19]Division of Disaster and Emergency Medicine, Department of Surgery Related, Kobe University Graduate School of Medicine, Kobe, Japan

[20]Emergency and Critical Care Medicine, Saga University Hospital, Saga, Japan

[21]Department of Emergency and Critical Care Medicine, Keio University School of Medicine, Tokyo, Japan

[22]Department of Emergency and Critical Care Medicine, Aizu Chuo Hospital, Aizu, Japan

[23]Emergency & Critical Care Center, Kawasaki Municipal Hospital, Kawasaki, Japan

[24]Advanced Medical Emergency & Critical Care Center, Yamaguchi University Hospital, Ube, Japan

[25]Center Hospital of the National Center for Global Health and Medicine, Tokyo, Japan

[26]Department of Emergency and Critical Care Medicine, Nippon Medical School, Tokyo, Japan

[27]Advanced Critical Care Center, Aichi Medical University Hospital, Nagakute, Japan

[28]Department of Emergency & Critical Care Medicine, School of Medicine, Kurume University, Kurume, Japan

[29]Department of Emergency Medicine, Trauma and Resuscitation Center, Teikyo University School of Medicine, Tokyo, Japan

[30]Department of Trauma, Critical Care Medicine, and Burn Center, Japan; Community Healthcare Organization, Chukyo Hospital, Nagoya, Japan

[31]Department of Acute and Critical Care Medicine, Sapporo Higashi Tokushukai Hospital, Sapporo, Japan

***Correspondence**: Takeshi Wada, MD, PhD

Division of Acute and Critical Care Medicine, Department of Anesthesiology and Critical Care Medicine, Hokkaido University Faculty of Medicine, N15, W7, Kita-ku, Sapporo Japan.

Tel: +81-11-706-7377

Fax: +81-11-706-7378

E-mail: twada1@med.hokudai.ac.jp

Supplementary Table S1. Baseline clinical characteristics of the patients in their 50s who did or did not receive anticoagulant therapy

N=108	Non-anticoagulant group N=75	Anticoagulant group N=33	*P*
Patient characteristics			
Age, years	54 (52–57)	54 (52–56)	0.353
Sex (female/male)	33.3/66.7 (25/50)	30.3/69.7 (10/23)	0.757
Preexisting conditions			
Charlson Comorbidity Index	0 (0–2)	1 (0–2)	0.885
ADL dependent/independent	9.3/90.7 (7/68)	21.2/78.8 (7/26)	0.120
Malignant disease, no/yes	85.3/14.7 (64/11)	81.8/18.2 (27/6)	0.644
Severe liver disease, no/yes	97.3/2.7 (73/2)	93.9/6.1 (31/2)	0.584
Prescribed anticoagulants, no/yes	88.0/12.0 (66/9)	97.0/3.0 (32/1)	0.278
Illness severity			
APACHE II score	19 (14–24)	29 (23–33)	<0.001
SOFA score	8 (5–11)	11 (9–13)	<0.001
SIRS score	3 (2–4)	3 (3–4)	0.093

ISTH DIC score	2 (1–4)	4 (3–6)	0.001
JAAM DIC score	3 (2–5)	5 (3–7)	<0.001
Septic shock, no/yes	41.3/58.7 (31/44)	15.2/84.8 (5/28)	0.008
Blood culture, negative/positive	44.0/56.0 (33/42)	54.5/45.5 (18/15)	0.312
Primary site of infection			0.365
Abdomen	17.3 (13)	24.2 (8)	
Lung	34.7 (26)	18.2 (6)	
Urinary tract	20.0 (15)	12.1 (4)	
Skin/soft tissue	14.7 (11)	24.2 (8)	
Blood stream	1.3 (1)	3.0 (1)	
Bone/joint	2.7 (2)	0 (0)	
CNS	1.3 (1)	3.0 (1)	
Endocardium	2.7 (2)	0 (0)	
Implant device	0 (0)	0 (0)	
Wound	1.3 (1)	3.0 (1)	
Others	4.0 (3)	12.1 (4)	
Therapeutic interventions			

Mechanical ventilation	52.1/47.9 (38/35)	45.5/54.5 (15/18)	0.523
PMX-DHP	97.3/2.7 (73/2)	75.8/24.2 (25/8)	0.001
IVIg	86.7/13.3 (65/10)	42.4/57.6 (14/19)	<0.001
Protease inhibitor	94.6/5.4 (70/4)	81.8/18.2 (27/6)	0.088
CRRT	77.3/22.7 (58/17)	30.3/69.7 (10/23)	<0.001
Corticosteroids	76.0/24.0 (57/18)	51.5/48.5 (17/16)	0.012
Noradrenaline	37.3/62.7 (28/47)	9.1/90.9 (3/30)	0.003
Enteral nutrition	50.7/49.3 (38/37)	21.2/78.8 (7/26)	0.004

Data are presented as proportions (counts) for categorical variables and medians (interquartile ranges) for continuous variables. Anticoagulant therapy was defined as the administration of antithrombin, recombinant human thrombomodulin, or their combination in the present study. ADL, activities of daily living; APACHE, Acute Physiology and Chronic Health Evaluation; CNS, central nervous system; CRRT, continuous renal replacement therapy; DIC, disseminated intravascular coagulation; IVIg, intravenous immunoglobulin; ISTH, International Society on Thrombosis and Haemostasis; JAAM, Japanese Association for Acute Medicine; PMX-DHP, polymyxin B direct hemoperfusion; SIRS, systemic inflammatory response syndrome; SOFA, Sequential Organ Failure Assessment.

Supplementary Table S2. Baseline clinical characteristics of the patients in their 60s who did or did not receive anticoagulant therapy

N=254	Non-anticoagulant group N=162	Anticoagulant group N=92	*P*
Patient characteristics			
Age, years	66 (63–67)	66 (63–68)	0.744
Sex (female/male)	32.7/67.3 (53/109)	39.1/60.9 (36/56)	0.303
Preexisting conditions			
Charlson Comorbidity Index	1 (0–2)	2 (0–2)	0.372
ADL dependent/independent	17.3/82.7 (28/134)	18.5/81.5 (17/75)	0.811
Malignant disease, no/yes	89.5/10.5 (145/17)	80.4/19.6 (74/18)	0.044
Severe liver disease, no/yes	96.3/3.7 (156/6)	96.7/3.3 (89/3)	1.000
Prescribed anticoagulants, no/yes	90.7/9.3 (147/15)	88.0/12.0 (81/11)	0.495
Illness severity			
APACHE II score	20 (15–29)	25 (19–33)	0.006
SOFA score	7 (5–10)	10 (7–13)	<0.001
SIRS score	3 (2–3)	3 (3–4)	0.278

ISTH DIC score	2 (1–4)	4 (3–5)	<0.001
JAAM DIC score	3 (2–4)	5 (4–6)	<0.001
Septic shock, no/yes	46.9/53.1 (76/86)	17.4/82.6 (16/76)	<0.001
Blood culture, negative/positive	47.8/52.2 (77/84)	27.2/72.8 (25/67)	0.004
Primary site of infection			0.080
Abdomen	22.2 (36)	37.0 (34)	
Lung	34.6 (56)	18.5 (17)	
Urinary tract	13.0 (21)	16.3 (15)	
Skin/soft tissue	12.3 (20)	10.9 (10)	
Blood stream	9.3 (15)	1.1 (1)	
Bone/joint	1.9 (3)	2.2 (2)	
CNS	3.7 (6)	3.3 (3)	
Endocardium	1.2 (2)	3.3 (3)	
Implant device	0 (0)	0 (0)	
Wound	2.5 (4)	0 (0)	
Others	5.6 (9)	7.6 (7)	
Therapeutic interventions			

Mechanical ventilation, no/yes	62.7/37.3 (99/59)	57.6/42.4 (53/39)	0.231
PMX-DHP, no/yes	96.3/3.7 (156/6)	81.5/18.5 (75/17)	<0.001
IVIg, no/yes	88.3/11.7 (143/19)	52.2/47.8 (48/44)	<0.001
Protease inhibitor, no/yes	93.2/6.8 (151/11)	84.8/15.2 (78/14)	0.030
CRRT, no/yes	83.3/16.7 (135/27)	45.7/54.3 (42/50)	<0.001
Corticosteroids, no/yes	76.5/23.5 (124/38)	48.9/51.1 (45/47)	<0.001
Noradrenaline, no/yes	38.9/61.1 (63/99)	15.2/84.8 (14/78)	<0.001
Enteral nutrition, no/yes	53.1/46.9 (86/75)	47.8/52.2 (44/48)	0.392

Data are presented as proportions (counts) for categorical variables and medians (interquartile ranges) for continuous variables. Anticoagulant therapy was defined as the administration of antithrombin, recombinant human thrombomodulin, or their combination in the present study. ADL, activities of daily living; APACHE, Acute Physiology and Chronic Health Evaluation; CNS, central nervous system; CRRT, continuous renal replacement therapy; DIC, disseminated intravascular coagulation; IVIg, intravenous immunoglobulin; ISTH, International Society on Thrombosis and Haemostasis; JAAM, Japanese Association for Acute Medicine; PMX-DHP, polymyxin B direct hemoperfusion; SIRS, systemic inflammatory response syndrome; SOFA, Sequential Organ Failure Assessment.

Supplementary Table S3. Baseline clinical characteristics of the patients in their 70s who did or did not receive anticoagulant therapy

N=329	Non-anticoagulant group N=241	Anticoagulant group N=88	*P*
Patient characteristics			
Age, years	75 (72–77)	74 (72–76)	0.510
Sex (female/male)	35.7/64.3 (86/155)	38.6/61.4 (34/54)	0.622
Preexisting conditions			
Charlson Comorbidity Index	2 (0–3)	2 (1–3)	0.123
ADL dependent/independent	26.1/73.9 (63/178)	18.2/81.8 (16/71)	0.811
Malignant disease, no/yes	87.6/12.4 (211/30)	85.2/14.8 (75/13)	0.580
Severe liver disease, no/yes	98.3/1.7 (237/4)	98.9/1.1 (87/1)	1.000
Prescribed anticoagulants, no/yes	90.7/9.3 (217/24)	88.0/12.0 (80/8)	0.814
Illness severity			
APACHE II score	23 (18–31)	28 (22–35)	0.006
SOFA score	9 (5–12)	10 (7–13)	0.013
SIRS score	3 (3–3)	3 (2–4)	0.245

ISTH DIC score	2 (1–4)	4 (3–5)	<0.001
JAAM DIC score	3 (2–5)	5 (4–6)	<0.001
Septic shock, no/yes	42.7/57.3 (103/138)	26.1/73.9 (23/65)	0.006
Blood culture, negative/positive	43.2/56.8 (104/136)	34.1/65.9 (30/58)	0.266
Primary site of infection			0.013
Abdomen	17.8 (43)	38.6 (34)	
Lung	43.6 (105)	25.0 (22)	
Urinary tract	20.3 (49)	13.6 (12)	
Skin/soft tissue	7.5 (18)	8.0 (7)	
Blood stream	0.8 (2)	1.1 (1)	
Bone/joint	1.2 (3)	1.1 (1)	
CNS	0.4 (1)	0 (0)	
Endocardium	0.8 (2)	1.1 (1)	
Implant device	0.8(2)	1.1(1)	
Wound	0 (0)	0 (0)	
Others	6.6 (16)	10.2 (9)	
Therapeutic interventions			

Mechanical ventilation, no/yes	58.2/41.8 (138/99)	55.7/44.3 (49/37)	0.916
PMX-DHP, no/yes	96.7/3.3 (233/8)	80.7/19.3 (71/17)	<0.001
IVIg, no/yes	92.9/7.1 (222/17)	51.1/48.9 (45/43)	<0.001
Protease inhibitor, no/yes	94.2/5.8 (227/14)	85.2/14.8 (75/13)	0.009
CRRT, no/yes	77.8/22.2 (186/53)	54.5/45.5 (48/40)	<0.001
Corticosteroids, no/yes	73.4/26.6 (177/64)	52.3/47.7 (46/42)	<0.001
Noradrenaline, no/yes	39.4/60.6 (95/146)	23.9/76.1 (21/67)	0.009
Enteral nutrition, no/yes	56.0/44.0 (135/106)	48.9/51.1 (43/44)	0.290

Data are presented as proportions (counts) for categorical variables and medians (interquartile ranges) for continuous variables. Anticoagulant therapy was defined as the administration of antithrombin, recombinant human thrombomodulin, or their combination in the present study.

ADL, activities of daily living; APACHE, Acute Physiology and Chronic Health Evaluation; CNS, central nervous system; CRRT, continuous renal replacement therapy; DIC, disseminated intravascular coagulation; IVIg, intravenous immunoglobulin; ISTH, International Society on Thrombosis and Haemostasis; JAAM, Japanese Association for Acute Medicine; PMX-DHP, polymyxin B direct hemoperfusion; SIRS, systemic inflammatory response syndrome; SOFA, Sequential Organ Failure Assessment.

Supplementary Table S4. Baseline clinical characteristics of the patients in their 80s who did or did not receive anticoagulant therapy

N=308	Non-anticoagulant group<br>N=222	Anticoagulant group<br>N=86	*P*
Patient characteristics			
Age, years	84 (82–86)	84 (82–86)	0.656
Sex (female/male)	45.0/55.0 (100/122)	46.5/53.5 (40/46)	0.817
Preexisting conditions			
Charlson Comorbidity Index	1 (0–2)	2 (0–3)	0.125
ADL dependent/independent	34.7/65.3 (77/145)	26.7/73.2 (23/63)	0.182
Malignant disease, no/yes	85.6/14.4 (190/32)	84.9/15.1 (73/13)	0.876
Severe liver disease, no/yes	98.6/1.4 (219/3)	100.0/0 (86/0)	0.563
Prescribed anticoagulants, no/yes	92.8/7.2 (206/16)	88.4/11.6 (76/10)	0.211
Illness severity			
APACHE II score	21 (16–27)	28 (22–33)	<0.001
SOFA score	8 (5–11)	10 (8–12)	<0.001
SIRS score	3 (2–4)	3 (2–4)	0.363

ISTH DIC score	3 (2–5)	5 (3–6)	<0.001
JAAM DIC score	2 (1–4)	4 (3–5)	<0.001
Septic shock, no/yes	45.0/55.0 (100/122)	19.8/80.2 (17/69)	<0.001
Blood culture, negative/positive	39.8/60.2 (88/133)	38.4/61.6 (33/53)	0.801
Primary site of infection			0.063
Abdomen	23.9 (53)	34.9 (30)	
Lung	31.1 (69)	16.3 (14)	
Urinary tract	24.3 (54)	32.6 (28)	
Skin/soft tissue	6.3 (14)	9.3 (8)	
Blood stream	1.8 (4)	0 (0)	
Bone/joint	2.7 (6)	1.2 (1)	
CNS	1.8 (4)	3.5 (3)	
Endocardium	1.8 (4)	0 (0)	
Implant device	1.8(4)	0 (0)	
Wound	1.4 (3)	0 (0)	
Others	3.2 (7)	2.3 (2)	
Therapeutic interventions			

Mechanical ventilation, no/yes	65.3/34.7 (141/75)	53.5/46.5 (46/40)	0.049
PMX-DHP, no/yes	95.5/4.5 (212/10)	77.9/22.1 (67/19)	<0.001
IVIg, no/yes	94.1/5.9 (209/13)	65.5/34.5 (55/29)	<0.001
Protease inhibitor, no/yes	98.2/1.8 (218/4)	90.6/9.4 (77/8)	0.002
CRRT, no/yes	87.8/12.2 (194/27)	59.3/40.7 (51/35)	<0.001
Corticosteroids, no/yes	83.8/16.2 (186/36)	49.4/50.6 (42/43)	<0.001
Noradrenaline, no/yes	45.2/54.8 (100/121)	19.8/80.2 (17/69)	<0.001
Enteral nutrition, no/yes	57.7/42.3 (128/94)	60.5/39.5 (52/34)	0.654

Data are presented as proportions (counts) for categorical variables and medians (interquartile ranges) for continuous variables. Anticoagulant therapy was defined as the administration of antithrombin, recombinant human thrombomodulin, or their combination in the present study. ADL, activities of daily living; APACHE, Acute Physiology and Chronic Health Evaluation; CNS, central nerve system; CRRT, continuous renal replacement therapy; DIC, disseminated intravascular coagulation; IVIg, intravenous immunoglobulin; ISTH, International Society on Thrombosis and Haemostasis; JAAM, Japanese Association for Acute Medicine; PMX-DHP, polymyxin B direct hemoperfusion; SIRS, systemic inflammatory response syndrome; SOFA, Sequential Organ Failure Assessment.

Supplementary Table S5. Scoring system for disseminated intravascular coagulation (DIC) by the Japanese Association for Acute Medicine (JAAM)

---

1. Clinical conditions that may be associated with DIC

    1) Sepsis/severe infection (any micro-organism)

    2) Trauma/burn/surgery

    3) Vascular abnormalities

        - large vascular aneurysms
        - giant hemangioma
        - vasculitis

    4) Severe toxic or immunological reactions

        - snakebite
        - recreational drugs
        - transfusion reactions
        - transplant rejection

    5) Malignancy (except bone marrow suppression)

    6) Obstetric calamities

    7) Conditions that may be associated with SIRS

        - organ destruction (e.g. severe pancreatitis)
        - severe hepatic failure
        - ischemia/hypoxia/shock
        - heat stroke/malignant syndrome
        - fat embolism
        - rhabdomyolysis
        - other

    8) Other

---

2. Clinical conditions that should be carefully ruled out

A. Thrombocytopenia

1) Dilution and abnormal distribution

Massive blood loss and transfusion, massive infusion

2) Increased platelet destruction

ITP, TTP/HUS, HIT, drugs, viral infection, alloimmune

destruction, APS, HELLP, extracorporeal circulation

3) Decreased platelet production

Viral infection, drugs, radiation, nutritional deficiency (vitamin B12, folic acid), disorders of hematopoiesis, liver disease, HPS

4) Spurious decrease

EDTA-dependent agglutinins, insufficient anticoagulation of blood samples

5) Other

Hypothermia, artificial devices in the vessel

B. Prolonged prothrombin time

Anticoagulation therapy, anticoagulant in blood samples, vitamin K deficiency, liver cirrhosis, massive blood loss and transfusion

C. Elevated FDP

Thrombosis, hemostasis and wound healing, hematoma, pleural effusion, ascites, anticoagulant in blood samples, antifibrinolytic therapy

D. Other

----

3. The diagnostic algorithm for SIRS

1) Temperature > 38 °C or < 36 °C

2) Heart rate > 90 beats/min

3) Respiratory rate > 20 breaths/min or $PaCO_2$ < 32 torr (< 4.3 kPa)

4) White blood cell > 12,000 cells/mm^3, < 4,000 cells/mm^3, or 10% immature (band) forms

----

4. The diagnostic algorithm

	Score
SIRS criteria	
≥3	1
0-2	0
Platelet counts (10^9/L)	
<80 or more than 50% decrease within 24 h	3

≥80 <120 or more than 30% decrease within 24 h	1
≥120	0
Prothrombin time (value of patient/normal value)	
≥1.2	1
<1.2	0
Fibrin/fibrinogen degradation products (mg/L)	
≥25	3
≥10 <25	1
<10	0
Diagnosis	
Four points or more	DIC

SIRS, systemic inflammatory response syndrome; ITP, idiopathic thrombocytopenic purpura; TTP, thrombotic thrombocytopenic purpura; HUS, hemolytic uremic syndrome; HIT, heparin-induced thrombocytopenia; APS, antiphospholipid syndrome; HELLP, hemolysis, elevated liver enzymes, and low platelet; HPS, hemophagocytic syndrome; EDTA, ethylenediaminetetraacetic acid; FDP, fibrin/fibrinogen degradation products.
